# Fusing fluorescent proteins and ferritin for protein cage based lighting devices†

**DOI:** 10.1039/d4nr05261g

**Published:** 2025-03-26

**Authors:** Alba Sanz-Velasco, Marta Patrian, Mattia Nieddu, Boxuan Shen, Juan Pablo Fuenzalida Werner, Mauri A. Kostiainen, Rubén D. Costa, Eduardo Anaya-Plaza

**Affiliations:** a Department of Bioproducts and Biosystems, Aalto University 02150 Espoo Finland eduardo.anaya@aalto.fi; b Technical University of Munich, Campus Straubing for Biotechnology and Sustainability, Chair of Biogenic Functional Materials Schulgasse 22 94377 Straubing Germany ruben.costa@tum.de; c Department of Medical Biochemistry and Biophysics, Karolinska Institutet 17177 Stockholm Sweden

## Abstract

Ferritin cages are an effective platform to encapsulate and stabilize a range of active cargoes and present a promising stepping stone towards a wide range of applications. They have been explored for optoelectronic applications in combination with fluorescent proteins towards bio-hybrid light-emitting diodes (Bio-HLEDs) only recently. However, protein integration within the cage or coassembled ferritin cages relies on electrostatic interactions and requires the supercharging of the fluorescent protein that easily compromises functionality and stability. To address this limitation, we have developed a fusion protein combining the *Thermotoga maritima* apoferritin (TmaFt) with a green fluorescent protein named mGreenlantern (mGL). This approach avoids jeopardizing both the cage assembly capability of TmaFt and the photophysical features of mGL. After optimizing the fusion protein mGL-TmaFt with respect to the linker length, assembling efficiency, and mGL payload into the cage (mGL@TmaFt), our findings reveal that they exhibited enhanced thermal and structural stabilities in both solution and when embedded into a polymer matrix. This enables effective mGL shielding, reducing H-transfer deactivation of the chromophore and water-assisted heat transfer across the polymer network. Indeed, the photo-induced heat generation in Bio-HLEDs operating at high currents was significantly reduced, resulting in a 30- and 15-fold higher device stability compared to references with either mGL or mGL-TmaFt proteins, respectively. Overall, this work sets in the potential of protein cage design for photon manipulation in protein lighting devices.

## Abstract 2

A novel approach towards bio-hybrid light-emitting diodes (Bio-HLEDs) is developed, by encapsulating a green fluorescent protein within an apoferritin cage. This renders up to 30-fold higher device stability compared to the fluorescent protein alone.

## Graphical abstract

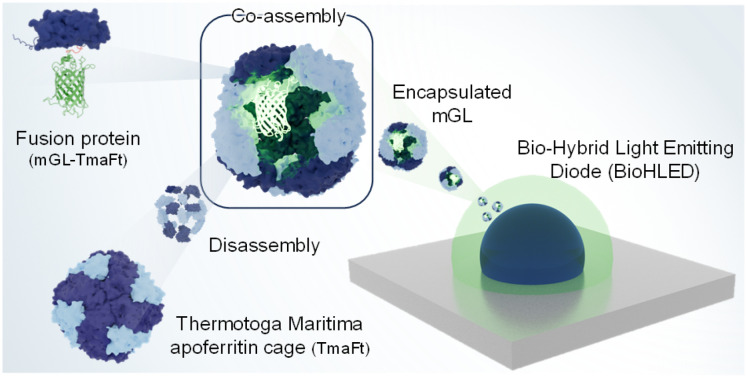

## Introduction

Protein cages are sophisticated supramolecular micro-compartments that self-assemble into regular architectures for the encapsulation of both bioinspired and man-made cargoes within their inner cavity.^1,2^ In nature, they can be found not only in the most common spherical-shaped structures, but also adopting vault^3^ and rod-like^4^ morphologies. Their well-defined geometry,^1^ non-toxicity,^2^ biodegradability and tendency to form crystalline arrays^5–7^ make them suitable for drug delivery,^8,9^ cell targeting^10^ and, more recently, optoelectronics.^11^ Additionally, their ability to entrap cargo limits its free diffusion toward the bulk solution, rendering chemically differentiated environments isolated from the surrounding media.^12^ Consequently, the local microenvironment within the shells exhibits unique properties, such as higher local concentration or variations in the pH.^13,14^ In addition, the cages increase the stability of the cargo by providing protection in a biologically relevant environment, *e.g.* against protease degradation.^15,16^ Therefore, protein cages have been successfully employed as carriers of different moieties, such as organic dyes,^17^ inorganic nanoparticles,^18^ and proteins.^19^ Finally, protein building blocks can be precisely modified by either chemical^20,21^ or genetic^22^ methods to enhance their native properties, realizing different functionalities with an exceptional versatility.

**Fig. 1:**
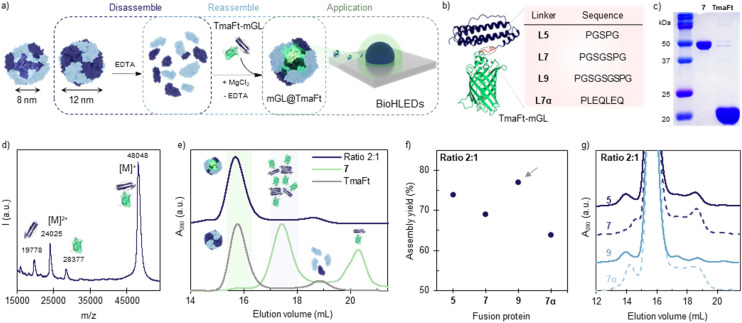
Design and characterization of fused mGL–TmaFt and its encapsulation within TmaFt protein cages. (a) TmaFt structure (PDB ID: 1VLG) and schematic representation of the TmaFt disassembly (with EDTA) and reassembly (with Mg^2+^) process, in the presence of TmaFt–mGL fusion protein to form mGL encapsulated cages and their application in Bio-HLEDs. (b) AlphaFold2-optimized structure of mGL–L*x*–TmaFt, highlighting in red the linker (L) connecting the TmaFt (blue) and mGL (green). The table displays the designed linkers and their corresponding amino acid sequences. (c) SDS-PAGE gel of mGL–L7–TmaFt (7) and TmaFt, highlighting the covalent nature of the construct. (d) Mass spectrum (MALDI-TOF) of construct 7, confirming the correct molecular weight (*M*_w_) of the fused mGL-TmaFt. (e) SEC of the 7@TmaFt cage at a ratio of 2 : 1 (blue). Peaks at 15.7 and 17.6 mL correspond to the assembled cages and non-assembled mGL–L7–TmaFt, respectively. Control samples 7 and the assembled TmaFt cage are shown in green and grey, respectively. Peaks at 17.5 mL and 20.4 mL correspond to aggregated and non-aggregated, respectively, 7. (f) Assembly yield calculated as the percentage of Abs_503_ in the main peak from SEC compared to the Abs_503_ in the whole sample at ratio 2 : 1. The best candidate has been marked with a grey arrow. (g) Zoom in on the aggregates’ retention time on the SEC chromatogram for the ratio 2 : 1 for all linkers.

Apoferritin (aFt), the iron-free form of ferritin, is one of the most recurrent protein cages in nature, as it can be found across organisms, ranging from archaea, bacteria, algae, plants and animals. Apoferritins have been extensively studied during the last few decades given their high thermal and chemical stability.^20,23^ The hollow cages show a 12 nm spherical structure, with an inner compartment of 8 nm (Fig. 1a).^24^ The disassembly and self-assembly processes are typically triggered by high ionic strength,^25^ the presence of chaotropic agents, such as guanidinium salts,^26^ and/or highly acidic or basic pH.^27^ Among the wide variety of aFt variants, *Thermotoga maritima* apoferritin (TmaFt, PDB ID: 1VLG; *M*_w_ of *ca.* 460 kDa)^25^ assembles in octahedral cages based on divalent cation (Mg^2+^) bridges between 12 dimers (24 monomers per cage), rendering very mild disassembly conditions based on Mg^2+^-sequestering and subsequent salt-mediated reassembly (Fig. 1a). This enables the integration of delicate cargoes, such as fluorescent proteins (FPs)^18^ and enzymes^28,29^ for application in medicine, photonics, and optoelectronics.

Regarding the latter, a recent example is the so-called bio-hybrid light-emitting diodes (Bio-HLEDs). They consist of using a high-energy emitting LED pumping chip covered by a color down-converting filter based on FPs embedded into, for example, a polymer or epoxy matrix.^30–35^ They promise to substitute rare earth and/or toxic down-converting materials applied to commercial light-emitting diodes (LEDs), since they are, indeed, compromising the sustainability of this technology in the long-term as noted by solid-state roadmaps.^36^ A main challenge in this field consists of identifying strategies to mitigate the photo-induced heat generation in high-power Bio-HLEDs, since this leads to fast FP degradation and thus, short device lifetimes. Today, Bio-HLEDs have achieved efficiencies above 120 lm W^−1^ and stabilities of either thousands of hours or a few hours at low and high applied currents, respectively.^31,37–39^ Among the different FP stabilization approaches, we have recently demonstrated the assets of using protein–protein systems based on, for example, supercharged FP-aFt cocrystals assembled *via* electrostatic self-assembly.^11^ Here, the device working temperatures were significantly reduced by entrapping the FPs in the aFt crystal lattice, enabling improved device stabilities by 40-fold.

Encouraged by the promising results yielded by protein–protein stabilization approaches for Bio-HLEDs, we explore FP-loaded protein cages as down-converting materials. The most common strategy to load foreign proteins into aFts relies on electrostatic complementarity between the negatively charged aFt cavity and a positively designed protein. However, this method often requires genetic manipulation to supercharge the cargo protein, which carries a significant risk of reducing its functionality and/or expression levels.^28,40^ On the other hand, only a few references (Table S1†) explored the expression of FPs fused with the protein cage monomer as the loading strategy. Previous research has primarily focused on characterising the concept of encapsulating foreign cargoes, by using FPs as a reporter to track the process. However, a comprehensive study of the photophysical properties of encapsulated FPs and their potential application in optoelectronics has not yet been conducted. Herein, we demonstrate that (i) the direct fusion of the TmaFt subunit with an archetypal green-emitting FP named mGreenLarten (mGL) is feasible and tunable using random linkers of different lengths (mGL–TmaFt), (ii) co-assembly of native and fusion subunits yields successfully encapsulated cages (mGL@TmaFt), and (iii) the use of mGL@TmaFt protein cages in Bio-HLEDs is an effective and novel concept toward reducing photon-induced heat generation, enhancing the overall performance.

Briefly, we optimize the length of the linkers to meet high assembly yield and mGL payload per cage, without affecting the native photophysical and thermal features of mGL. Size exclusion chromatography (SEC), dynamic light scattering (DLS), microscopy, thermocycling, and steady-state as well as time-resolved spectroscopy support this optimization. Finally, the best mGL@TmaFt protein cages were implemented in a cellulose-based color down-converting filter for high-power Bio-HLEDs that featured a significant reduction of the working temperature down to 40 °C, realizing a 30- and 15-fold higher stability compared to reference devices with mGL and the fusion protein mGL–TmaFT, respectively. Overall, this work sets in a successful strategy of fusing FPs and aFt subunits in protein cages as an effective concept toward zero-thermal-quenching color down-converting materials for stable protein-based lighting applications.

## Results and discussion

### Linker design of the fusion protein mGL–TmaFt

The direct fusion of domains can often lead to protein misfolding,^41^ low expression yield,^42^ or impaired bioactivity.^43^ In this context, natural multidomain protein studies have shown that a high successful rate is obtained with flexible linkers bearing an average length of 6.5 residues^44^ with threonine (T), serine (S), proline (P), and glycine (G) heavily repeated.^45^ Thus, a conservative design was chosen with five, seven, and nine residue flexible linkers – *i.e.*, PGSPG (L5), PGSGSPG (L7), and PGSGSGSPG (L9), as shown in Fig. 1b and S1.† An additional linker (PLEQLEQ, L7α) showing rigidity due to its alfa-helix morphology was prepared. Genes encoding the engineered fusion proteins (mGL–TmaFt) and the protein cages (TmaFt) were successfully cloned and expressed in *E. coli*, as well as purified by affinity chromatography (197 mg protein in 1 L of culture). The purified proteins were characterized by SDS-PAGE gel electrophoresis and MALDI-TOF mass spectroscopy, confirming the successful coupling. As an example, Fig. 1c and d show the above characterization of the construct mGL–L7–TmaFt (7) encoded by the sequence containing L7, while Fig. S2 and S3† display the same information for the other constructs mGL–L5–TmaFt (5), mGL–L9–TmaFt (9) and mGL–L7α–TmaFt (7α), respectively. As an example among the series, SDS-PAGE gel electrophoresis shows a single band at *ca.* 50 kDa for 7 and *ca.* 20 kDa for TmaFt cages, while mass spectroscopy of 7 shows a main peak at 48 048.24 g mol^−1^, matching well with the calculated mass of 48 179 g mol^−1^ (0.2% difference is related to *e.g.* amino acid deletion). The additional peaks shown at 19 778 and 28 377 g mol^−1^ are assigned to the constituent proteins TmaFt and mGL, respectively, as they split upon ionization. Finally, the photophysical properties of mGL–L*x*–TmaFt proteins remain unchanged compared to native mGL, showing no spectral emission changes associated with photoluminescence quantum yields *ϕ* of 70–75% and excited state lifetimes *τ*_450_ of around 3.2 ns, Table S2 and Fig. S4.†

### mGL encapsulation *via* cage self-assembly in mGL@TmaFt

The cage self-assembly was achieved by adapting the previously reported methodology.^46^ Briefly, TmaFt protein cages (2.5 mg) were disassembled by adding up to 100 mM EDTA in 20 mM pH 8.1 Tris buffer. The resulting protein dimers were then mixed with the selected fusion protein (5, 7, 7α, and 9) at 2 : 1 mGL–TmaFt : TmaFt (fusion protein/native cage ratio). To trigger their statistical self-assembly, the mixtures were dialyzed against 50 mM MgCl_2_, 20 mM pH 8.1 Tris buffer, while the encapsulation efficiency was assessed by size exclusion chromatography (SEC, Fig. 1e and S5, S6†). The chromatograms show a major peak at 15.7 mL corresponding to the self-assembled mGL@TmaFt cage. This assignment is based on the native TmaFt control (Fig. 1e, grey), showing two peaks at 15.7 and 18.9 mL corresponding to the TmaFt cage and TmaFt protein dimer, respectively. The visually observed green colour suggests the presence of mGL protein in the mGL@TmaFt fraction, as confirmed by the absorption peak at 503 nm – Fig. S5.† The encapsulation efficiency was calculated by measuring the protein content (by absorbance at 280 nm) in the mGL@TmaFt fraction at 15.7 mL compared to the total protein content (peaks at 15.7, 17.5, 18.9, and 20.3 mL), Fig. 1f and S6. It shows a good encapsulation yield for all the fusion proteins with flexible linkers (between 70–77%, with 9 being the highest), indicating a limited effect of the linker length in the process, Fig. 1f and g. On the other hand, the rigid linker 7α shows a lower yield (64%), highlighting the positive effect of flexible linkers that allow better dynamic rearrangement of cage moieties during assembly.

**Fig. 2:**
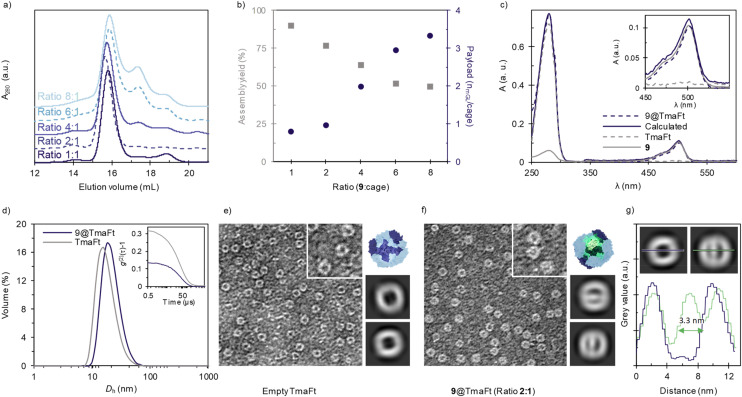
Characterization of 9@TmaFt cages by different techniques. (a) SEC chromatogram of 9@TmaFt at different 9 : TmaFt ratios. (b) Payload (blue dots) calculated as the number of mGL per cage and assembly yield (grey squares) at increasing ratio. (c) Normalized absorbance for a concentration of 1 μM. Inlet: zoom in of Abs (1 μM) between *λ*_450_ and *λ*_550_. More detailed explanation can be found in ESI Fig. S7.† (d) Volume-averaged particle size distribution measured by DLS of 9@TmaFt encapsulated in a 2 : 1 ratio, and TmaFt closed cages in blue and grey lines, respectively. Inset: second order normalized autocorrelation function of both samples. Negative-stain TEM images of (e) empty TmaFt cages and (f) 9@TmaFt encapsulated in a 2 : 1 ratio. The image width in both cases corresponds to 200 nm. Next, model and images of two representative 2D class average images of (e) empty TmaFt protein cages and (f) loaded TmaFt obtained using CryoSPARC software. (g) Mean grey value profile *vs.* distance for empty and 9@TmaFt (blue and green respectively, inset).

Since lighting applications require a higher protein content, we evaluated the luminescent protein-cage formation at different mass ratios of 9 : TmaFt cage ratios 1 : 1, 2 : 1, 4 : 1, 6 : 1, and 8 : 1, Fig. 2a. Unfortunately, the increase of mGL–TmaFt ratio linearly reduces the efficiency loading down to 50% for 6 : 1, followed by a plateau at higher mass ratios, Fig. 2b, grey squares. Indeed, two additional peaks in the SEC analysis appear at (i) 17.6 mL, corresponding to the fused protein oligomers formed in the presence of Mg^2+^ as seen in the corresponding control (Fig. 1e, solid green), and (ii) 20.3 mL that is assigned to the presence of monomeric mGL–TmaFt. Thus, the natural tendency of mGL–TmaFt to aggregate (Fig. 1e, solid green) and the steric hindrance that a high payload may cause during the encapsulation process are detrimental if the cargo to cage ratio is increased.

Indeed, the number of FPs per cage also increases at high mass ratios, Fig. 2b. This was determined by monitoring absorption changes at relative intensities of the bands centered at 280 nm (all proteins present in the cargo and shell) and 503 nm (only corresponding to the cargo mGL) of each sample after SEC purification, Fig. 2c and S7 for detailed calculations. As expected, at low ratios like 1 : 1 and 2 : 1, an average of one mGL is encapsulated per cage, while higher ratios render payloads with a maximum of three mGLs, Fig. 2b. This is in accordance with the maximum payload volumes achieved by other experimental methods based on electrostatic encapsulation.^46^ Overall, 9 shows the best compromise between optimal encapsulation, showing a maximum efficiency yield of 77% and one mGL loading at 2 : 1 ratio, allowing us to further proceed without further purification steps and to provide a fair comparison in Bio-HLEDs – *vide infra*. Unfortunately, ratios higher than 2 : 1 that result in higher payloads could not be further pursued for applications. The increased yield of fusion-protein oligomers and subsequent reduction in encapsulation yield would require a purification step (*e.g.* SEC). It would limit the downstream production process, yielding insufficient protein material for their application in BioHLEDs.

### Characterization of mGL@TmaFt cages

Once the linker and the mGL–TmaFt/TmaFt cage ratio were optimized – *i.e.*, 9, in a 2 : 1 ratio to the TmaFt cage, 9@TmaFt was obtained. The structural and emission features for the cages are characterized by DLS, microscopy, and steady-state and time-resolved emission spectroscopy techniques. DLS shows a single peak with a volume-average hydrodynamic diameter of 22 nm, which agrees with the diameter of native TmaFt (18 nm), Fig. 2d. Thus, there is no large variation in the particle hydrodynamic diameter between empty and loaded cages, supporting the above results obtained by SEC. It also supports that the mGL has been successfully encapsulated inside the protein cage and is not pointing outwards the cage. Further confirmation was obtained by visualizing samples with and without cargo using TEM, Fig. 2e, f and ESI.† Here, TmaFt is shown as empty spherical particles with a dark core derived from heavy atom staining, while 9@TmaFt shows a clearer core, indicating the presence of protein within the cage, Fig. 2f, inset and Fig. S8–S10.† A more detailed image analysis using CryoSPARC – Fig. 2f – supports the previous hypothesis. Indeed, the 2D classification of the particles picked from the TEM images (see the ESI† for more details) shows that the presence of a significant portion of cages has encapsulated the cargo. The diameter was determined as 13.3 nm for all cages, highlighting no structural distortion when loaded. In addition, mean grey value profiles were measured for selected 2D class average images of empty TmaFT and 9@TmaFt across an equatorial section, Fig. 2g, giving an estimated diameter for the mGL beta barrel of 3.3 nm, in good agreement with the previously reported data of closely-related fluorescent proteins.^47^

**Fig. 3:**
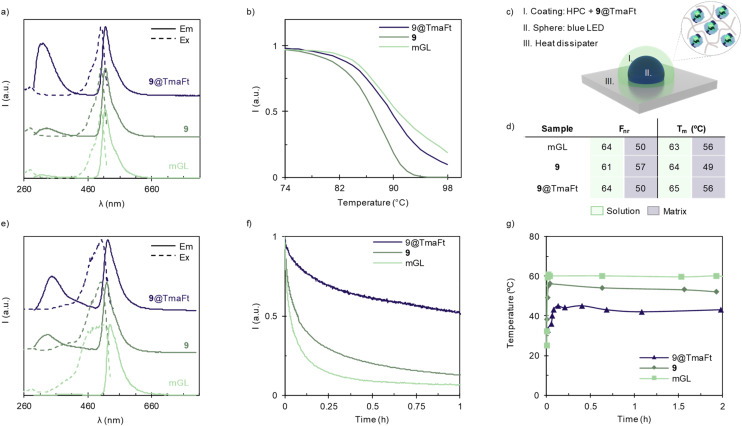
Characterization of Bio-HLEDs formed by 9@TmaFt immobilized in HPC. (a) Excitation (dashed line) and emission (solid line) spectra in solution of 9@TmaFt in 2 : 1 ratio (blue), 9 (dark green) and mGL (light green), measured at an emission wavelength of 520 nm and excitation wavelength of 280 nm, respectively. (b) Temperature of non-reversibility (*T*_nr_) in solution of 9@TmaFt in 2 : 1 ratio, 9, and mGL. (c) Bio-HLED schematic. (d) Overview of refolding capability (*F*_nr_) and melting temperature (*T*_m_) in solution (green) and in matrix (dark blue). Values are calculated at 50% of the emission intensity loss for *T*_m_. (e) Excitation (dashed line) and emission (solid line) spectra of 9@TmaFt in 2 : 1 ratio (blue), 9 (dark green) and mGL (light green) in HPC, measured at an emission wavelength of 520 nm and an excitation wavelength of 280 nm, respectively. (f) Device stability highlighted by the emission intensity decay of the 9@TmaFt coating over time operating under high power conditions (150 mW cm^−2^). (g) Device working temperature changes of the 9@TmaFt coating over time operating under high power conditions (150 mW cm^−2^).

Finally, the photoluminescence figures-of-merit of 9@TmaFt are comparable to those of the respective references 9 and native mGL in solution, Fig. 3a. The excitation spectrum for 9@TmaFT does not show differences compared to 9 and native mGL in the chromophore region, while a strong intensity increase of the band centered at 330 nm is related to the higher contribution of aromatic moieties present in the protein cages, Fig. S11.† Indeed, the emission spectra show the same shape associated with the same *ϕ* and *τ* values as those of the references, Fig. 3a and Table S2,† suggesting that the FP conformation remains intact and is not significantly distorted upon protein encapsulation. This is also confirmed by the absence of any emission features of the neutral form of the protein chromophore in the 400–470 nm region, Fig. 3a.^48^ These findings are also valid for 5@TmaFT, 7@TmaFT and 7α@TmaFT constructs, Table S2 and Fig. S11.† Finally, thermal features in solution were also studied by modulated scanning fluorimetry assays, Fig. 3b and S12.† Here, 9 shows a lower refolding capability (*F*_nr_), Fig. 3d green columns, pointing out a decrease in stability compared to the native mGL. However, 9@TmFt shows similar thermal dynamic behaviour to mGL, highlighting the relevant role of protein cages in the stabilization of FPs.

### 9@TmaFt cages applied to Bio-HLEDs

As a final step, we fabricated colour filters embedding the target 9@TmFt into hydroxypropyl-cellulose (HPC) coatings following the procedure, see the ESI† for more details.^49,50^ As references, 9 and native mGL proteins were similarly implemented at the maximum mGL amount of 0.25 mg per coating. This protein loading is unfortunately limited by the production yield of 9@TmaFt and its colloidal stability at higher concentrations. All the produced coatings were homogeneous to the naked eye. The thermal features of the coatings were also studied by modulated scanning fluorimetry assays, Fig. 3d blue columns and Fig. S13.† Here, the 9@TmFt films show a lower *F*_nr_ but higher melting temperature (*T*_m_) than those of the 9 coatings, highlighting the effective isolation of the mGL inside the protein cage. As noted in the literature, the protein cage provides a robust shield for the cargo from the surrounding environment, significantly reducing H-transfer events and/or water molecule and ion exchange, thereby allowing their use, for example, as contrast agents for magnetic resonance imaging.^51–54^ Thus, it is not surprising that the thermodynamic stability of mGL inside the protein cage is enhanced because of the crowding effects on protein–protein interactions that are expected to reinforce the H-bonding network in the mGL scaffold surrounding.^55^ Indeed, the thermal behaviour of the aggregated mGL coatings is similar to that of 9@TmFt coatings, supporting the above statement. As a further confirmation, the excitation spectra show a well-defined structure for 9@TmaFt and 9 coatings, while a broad featureless band for mGL films is noted, Fig. 3e. In addition, the mGL aggregation in HPC coatings arises from the strongly red-shifted emission (20 nm) and the increase of its fluorescence lifetime: it shows a *τ*_450_ of 4.8 ns, in stark contrast to those of 9 and 9@TmaFt in coatings (3.7 and 3.5 ns, respectively), as well as mGL in solution (3.2 ns), Fig. 3a and Table S2.† These findings suggest that (i) the presence of TmaFt is effective in preventing protein agglomeration upon film formation (9*vs.* mGL), and (ii) a lack of aggregation in both 9@TmaFt and 9 coatings will enable us to provide a fair comparison to determine the impact of the protein encapsulation on the device performance. Regardless of the aggregation behaviour, the *ϕ* value of around 66% is met for all the coatings, Table S2.†^51–55^

Finally, Bio-HLEDs were prepared with blue-emitting LEDs (450 nm; Winger Electronics, 1 W) directly covered by the above 9, 9@TmFt, and mGL coatings and were driven at a high applied current of 200 mA or a photon power density of 150 mW cm^−2^, Fig. 3c. All the films showed a similar partial conversion of the blue LED emission band to the expected green emission band of the down-converting material, but a very different stability and thermal behaviour, Fig. 3f and g. In short, mGL- and 9-based devices exhibited an exponential emission decay of the green emission band intensity going together with the increase of the coating temperature up to 60 ± 2 and 56 ± 2 °C, respectively. Thus, the device stability (time to reach 50% loss of the initial intensity of the conversion band) is ruled by the emission temperature quenching, reaching values of around 4 min for 9 and 2 min for mGL devices. Interestingly, though the photo-induced heat generation is similar for both devices, the exponential decay of 9 is much less pronounced, requiring up to 1 h to reach a complete disappearance *e.g.*, mGL devices needs 25 min; Fig. 3f. This suggests that the TmaFt moiety might effectively prevent the H-transfer events around the protein scaffold that typically leads to the deactivation of the mGL chromophore.^31,37^ Much more striking is the finding that the 9@TmaFt shows a much milder exponential decay as the maximum working temperature is effectively reduced to 42 ± 2 °C, Fig. 3f and g. Thus, the device stability increases up to 1 h (*i.e.*, 30- and 15-fold higher stability compared to mGL or 9 devices, respectively) and 4.5 h (extrapolated) for a complete emission quenching that is average top compared to the prior-art Bio-HLEDs.^11,37,38,56^ These results indicate that encapsulating FPs in ferritin cages provides a more efficient blocking barrier for the water-assisted heat transfer process from the FP to the polymer network than that of the uncontrolled aggregates (mGL *vs*. 9@TmFt devices). Additionally, it protects from the H-transfer process that renders the non-emissive neutral form of the photosensitizer within the FP (9@TmFt *vs*. 9/mGL devices).

## Conclusions

This work addresses the challenge of improving the thermal stability of FPs for their use in Bio-HLEDs using protein cages. To this end, we have designed a family of fusion proteins combining mGL and TmaFt with linkers of different lengths to explore their influence during encapsulation (mGL@TmaFt). Here, we have optimized them with respect to high assembly yield and mGL cargo amount without affecting the photoluminescence and thermal features compared to those of the native mGL. The best conditions to obtain a 77% assembly yield with one mGL per cage were achieved with the larger linker (L9), as corroborated by SEC, DLS, TEM, and time-resolved and steady-state emission spectroscopy of the 9@TmaFt cages in solution. Furthermore, the photoluminescence and thermal features were also preserved in HPC-based coatings, enhancing the device performance of high-power Bio-HLEDs. They showed a significant reduction of the working temperature down to 42 ± 2 °C compared to reference devices with mGL and 9 coatings (*ca*. 60 °C). Thus, the device stability is 30- and 15-fold higher compared to the respective references, while the overall stability of *ca*. 1 h is comparable to the state-of-the-art high-power Bio-HLEDs.^11,37,38,56^ Overall, this work expands the potential of protein cages to encapsulate a wider range of cargoes with or without net charge by protein engineering and a highly modular encapsulation (a simple statistical mixture of recombinant and fused TmaFt in solution) toward advancing this optoelectronic field. Further research on designing protein cages with multi-component FPs as well as the control of the inner protein–protein interactions would enable multi-colour protein cage-based lighting devices with increased stability.

## Author contributions

A. S. V. conducted molecular biology, protein purification, cell culture work and structural characterization (spectroscopy, DLS, TEM). M. P. planned protein engineering. M. N. performed device fabrication and characterization. B. S. performed particle picking analysis. Project and experimental design and data analysis: J. P. F.-W., M. A. K., R. D. C., and E. A. P. A. S. V., R. D. C. and E. A. P. wrote the manuscript. All the authors revised the text.

## Conflicts of interest

There are no conflicts to declare.

## Supplementary Material

NR-017-D4NR05261G-s001

## Data Availability

The data supporting this article have been included as part of the ESI.†
