# Supplementary material for: Fusing fluorescent proteins and ferritin for protein cage based lighting devices
Source: Nanoscale. 2025 Mar 26;17(17):10793–800. doi: 10.1039/d4nr05261g (PMC11970472; doi:10.1039/d4nr05261g)
Supplement: NR-017-D4NR05261G-s001 [file NR-017-D4NR05261G-s001.pdf]

Supplementary Information (SI) for Nanoscale.
This journal is © The Royal Society of Chemistry 2025

Supporting Information

Fusing Fluorescent Proteins and Ferritin for Protein Cage Based Lighting Devices

Alba Sanz-Velasco,[a] Marta Patrian,[b] Mattia Nieddu,[b] Boxuan Shen,[c] Juan Pablo Fuenzalida Werner,[b] Mauri A. Kostiainen,[a] Rubén D. Costa,[b] and Eduardo Anaya-Plaza[a]**

[a] Department of Bioproducts and Biosystems, Aalto University, 02150 Espoo (Finland). Email: eduardo.anaya@aalto.fi

[b] Technical University of Munich, Campus Straubing for Biotechnology and Sustainability, Chair of Biogenic Functional Materials, Schulgasse 22, 94377, Straubing (Germany). Email: ruben.costa@tum.de

[c] Department of Medical Biochemistry and Biophysics, Karolinska Institutet, Stockholm 17177, Sweden

Table of Content

Methods S3
Chemicals S3
Protein Production S3
Recombinant Protein Purification S3
Encapsulation Essay S3
Size Exclusion Chromatography (SEC) and UV-Vis Absorption Spectroscopy S4
Transmission Electron Microscopy S4
Particle picking S4
SDS-PAGE gels S4
Dynamic Light Scattering (DLS) S4
MALDI-TOF S4
Thermocycler-based Modulated Scanning Fluorimetry S5
HPC based Coatings S5
Device Characterization S5
Supplementary Information- Table S1 S6
Supplementary Information- Figure S1 S7
Supplementary Information- Figure S2 S7
Supplementary Information- Figure S3 S8
Supplementary Information- Table S2 S8
Supplementary Information- Figure S4 S9
Supplementary Information- Figure S5 S9
Supplementary Information- Figure S6 S10
Supplementary Information- Figure S7 S10
Supplementary Information- Figure S8 S11
Supplementary Information- Figure S9 S12
Supplementary Information- Figure S10 S13
Supplementary Information- Figure S11 S13
Supplementary Information- Figure S12 S14
Supplementary Information- Figure S13 S15
Supplementary Information- Figure S14 S16
References S17

Methods

Chemicals

All chemicals were purchased from Sigma-Aldrich and used without further purification, unless stated otherwise. Milli-Q grade water was used in all experiments. Main buffer was 20 mM Tris (Product No. T6066), 50 mM $MgCl_2$ (Product No. 1.05833) pH 8.1 for all experiments, unless stated otherwise.

Protein Production

All constructs used in this research were ordered as codon optimized synthetic genes (Twist Bioscience) cloned within bacterial expression vector pET29b(+). Proteins were under the control of the T7-LacO promoter and N-terminally tagged with 6x HisTag for subsequent protein purification. The protein plasmid constructs were transformed in electrocompetent *Escherichia coli* BL21 DE3 and plated on Luria Bertani (LB, purchased from Gerbu, Product No. 1417) agar plates, containing 50 µg/mL Kanamycin (Kan, Product No. K1377). Single colonies were picked, inoculated in LB medium, and grown shaking overnight at 30 °C. Following, certain volume of preculture was reinoculated in fresh LB with Kan to obtain an initial optical density (OD_{600}) of 0.1 and grown at 30 °C until reaching OD_{600} 0.4-0.6. At this point, protein production was induced with 1 mM of Isopropyl β-D-thiogalactopyranoside (IPTG, Product No. 5810) and the bacterial cells were incubated at 16 °C shaking (200 rpm) for 72 h.

Recombinant Protein Purification

Bacterial cells were harvested via centrifugation (30 min, 5000 g, 4 °C) and subsequently washed with PBS (NaCl 8 mg/L, KCl 0.2 g/L, Na_2HPO_4 1.42 g/L, KH_2PO_2 0.27 g/L, MQ water; pH 7.4) and centrifuged afterwards (20 min, 4000 g, 4 °C). Pellet was resuspended in PBS and kept on ice during sonication (amplitude of 80 and complete process time of 8 min). Disrupted cell suspension was centrifuged for 1.30 h at 41600 g and 4 °C. His-tagged proteins present in the supernatant were purified via HisTrap (Äkta pure Cytiva, flow 5 mL/min with a gradient from 0% - 100 % 0.5 M Imidazole, PBS in 6 CV) and fusion proteins were desalted via HiPrep 26/10 Desalting column (Äkta pure Cytiva, flow 20 mL/min in isocratic). TmaFt protein cages were purified *via* HisTrap and then, passed through a SEC (HiLoad 26/600 Superdex 75 pg, flow 2.5 mL/min) using 10 mM EDTA, PBS. Unicorn 6 software was used to evaluate the chromatography data. After purification, proteins were concentrated with Centrifugal Filter Units (Merck), frozen in liquid nitrogen, and stored at – 80 °C.

Encapsulation Essay

Before encapsulation all samples were firstly dialyzed against 20 mM Tris pH 8.1 buffer. Encapsulation was done by following the protocol described by Chakrabroti, S. *et al.*[S1] with minor modifications. TmaFt protein cages (2.5 mg host) were disassembled in presence of 100 mM EDTA (Product No. E6758). After incubation for 30 min at 4 °C and 600 rpm, fusion protein was added (*e.g.* 0.45 mg guest for the ratio 2:1 fusion protein/cage). Final volume in all experiments was adjusted to 500 µL. Then, samples were left for 30 min at 4 °C and 600 rpm. Finally, dialysis was carried out at 4 °C against 20 mM Tris, 50 mM $MgCl_2$ pH 8.1 buffer to remove EDTA and add Mg^{2+} thus, triggering cage assembly.

Size Exclusion Chromatography (SEC) and UV-Vis Absorption Spectroscopy

Encapsulation was assessed by SEC by passing the sample through a Superose 6 Increase 10/300 GL high resolution size exclusion column (Äkta pure Cytiva, flow 0.5 mL/min). Peaks were collected in different fractions for further characterized by UV-Vis absorption spectroscopy. All results were obtained using Tris 20 mM, $MgCl_2$ 50 mM pH 8.1 buffer, unless stated otherwise. Absorption spectrum was acquired for each fraction with a UV-Vis spectrometer UV-2600 (Shimadzu), using a wavelength range 220–800 nm, scan speed medium, threshold 0.01 and a slit width of 2.0. The data shown in the encapsulation experiments are from single data acquisition in interest of protein economy, as they were prepared in large scale and after purification they are not suitable for devices.

Transmission Electron Microscopy

TEM images were obtained using FEI Tecnai 12 microscope operated at an acceleration voltage of 120 kV. 3.5 µL of sample (0.1 mg/mL protein) solution was deposited on to glow-discharged copper-Formvar grid, incubated for 2 min and then, excess solution was removed by blotting with filter paper. Shortly after, 3.5 µL of uranyl formate (2%, purchased in Electron Microscopy Science, Product No. 22451) was added for staining, incubated for 40 s and finally excess stain was blotted away with filter paper.[S2] Images have been processed using ImageJ FIJI.

Particle picking

Negative-stained TEM images of 30K magnification were imported into CryoSparc software. An initial set of particles was picked with blob picker tool, then the picked particles were extracted and 2D classified. Then, using all the 2D classes clearly represent protein cages as template, the particles used for analysis were picked and extracted again from the original TEM images with template picker tool. Finally, the particles were 2D classified to generate the 2D class average images.

SDS-PAGE gels

12% acrylamide SDS-PAGE gels were prepared. Protein samples were mixed with 4x loading buffer containing DTT and SDS. They were heated for 5 min at 95 °C and then, centrifuged using a bench-top centrifuge. Samples were run and separated on a gel using a constant voltage of 200 V for approximately 1 h. Gels were stained using Coomassie blue.

Dynamic Light Scattering (DLS)

The hydrodynamic diameter (D_h) of cages was measured using a Malvern Instruments DLS device (Zetasizer Nano ZS Series) with a 4 mW He-Ne ion laser at a wavelength of 633 nm and an avalanche photodiode detector at an angle of 173 °. All experiments were carried at room temperature with PMMA cuvettes using 100 µL of sample at 0.1 mg/mL. Zetasizer software (Malvern Instruments) was used to obtain the particle size distribution.

MALDI-TOF

Sample (0.1 mg/mL) was mixed with the same volume of matrix (40 mg/mL sDHB, Product No. 50862, in TA30). Experiments were carried out with Bruker UltrafleXtreme TOF/TOF on a ground-steel plate and results were analyzed with the software FlexAnalysis 3.4.

Thermocycler-based Modulated Scanning Fluorimetry

Modulated Scanning Fluorimetry (MSF) was performed as described in Svilenov *et al.*[S3] MSF measurements were performed with Thermocycler CFX96 Touch Real-time PCR System (Bio-Rad). The program is based on heating and cooling cycles ranging from 25 °C to 99 °C to measure the progressive loss of fluorescence and the irreversible unfolding of the FPs. Samples were heated 5 °C per sec and held for 1 min at the temperature peak, followed by a recovery period of 5 min at 25 °C. FP concentration per well was 1 μM to avoid saturation. Thermograms were buffer-subtracted and normalized by the highest fluorescence read-out of each sample. Mean values and standard deviations of triplicate were calculated and plotted (Figures S12-S13). Melting curves were obtained plotting the fluorescence values obtained at peak temperatures, while non-reversibility curves were obtained plotting the fluorescence values obtained at 25 °C.

HPC based Coatings

The hydroxypropyl cellulose (HPC, Product No. 435007) coatings were prepared as follows. HPC was dissolved in Milli-Q water, with a concentration of 230 mg mL^{-1}. 500 μL of the HPC solution were mixed with 300 μL of the respective FP solution (9.26 nmol). The coating was obtained after stepwise drying of the solution by vacuum to form a dome-shaped coating.

The photophysical studies were carried out using a UV-vis spectrometer UV-2600 (Shimadzu), using a wavelength range 200–800 nm, scan speed medium, threshold 0.01 and a slit width of 2.0 and a FS5 Spectrofluorometer (Edinburgh Instruments) with the SC-05/SC-10 module for liquid/solid samples and the time-correlated single photo-counting or TCSPC (64.3 ps pulse width) module to determine excited state lifetime (τ). The data was then adjusted to a bi-exponential decay fit using Origin Software. To calculate the average lifetime for each FP-coating, the following equation was used[S4]

$$\tau = \frac{\int_0^x t\, \Sigma a_i \exp\left(-\frac{t}{\tau_i}\right) dt}{\int_0^x \Sigma a_i \exp\left(-\frac{t}{\tau_i}\right) dt} = \frac{\Sigma a_i {\tau_i}^2}{\Sigma a_i \tau_i}$$

where a_i (λ) is the amplitude fractions and τ_i are the lifetimes. The measurements were performed at room temperature. The photoluminescence quantum yield (ϕ) was measured using a Quantaurus-QY Absolute PL quantum yield spectrometer (Hamamatsu Photonics).

Device Characterization

The above coatings were directly placed on top of the commercial 1 W, Winger Electronics, 450 nm LED used without further modification. The Bio-HLEDs were characterized using a Keithley 2400 as a current source, while the changes in the electroluminescence spectrum were monitored using an AVS-DESKTOP-USB2 (Avantes) in conjunction with a calibrated integrating sphere Avasphere 30-Irrad. The changes in the FP-coating temperature were monitored using a thermographic camera T430sc (FLIR) coupled to the measuring systems. The device stability assays were reproduced two times as shown in Figure S14.

Supplementary Information- Table S1

Table 1. Summary of the studies on fluorescent protein encapsulation and the methodology employed.

Cage	Size (nm)	FP	Encapsulation method	FP stability characterization	SI Ref.
Bacillus subtilis Lumazine Synthase (BsLS)	16	GFP	Fusion to riboflavin synthase (BsRS)	No photophysical characterization. They reported an increase up to 15 times in fluorescence when encapsulated, although no calculation on payload.	S5
Qβ virus like particle	28	sfGFP	Rev-tag/α-Rev aptamer. Qβ genome packaging hairpin/CP	Identical absorbance and emission wavelengths, and rates of photobleaching as the free fluorescent proteins. Excitation and emission intensities of the packaged proteins more intense than free ones. Cargo more resistant to thermal denaturation in presence of 0.5 % of SDS.	S6
Aquifex aeolicus Lumazine Synthase (AaLS)	16	GFP	Fusion to riboflavin synthase (AaRS)	-	S7
Thermatoga maritima encapsulin	25	sfGFP	Fusion to encapsuling monomer	-	S8
Archaeoglobus fulgidus apoferritin	12	GFP	Fusion to aFt monomer	-	S9
Archaeoglobus fulgidus apoferritin	12	GFP (+36)	Electrostatic interaction	-	S10
Archaeoglobus fulgidus apoferritin	12	GFP (+36)	Electrostatic interaction	-	S11
Cowpea Chlorotic Mottle Virus (CCMV)	28	EGFP	K-coil/E-coil recognition	-	S12
Bacteriophage P22	60	GFP and mCherry	Fusion to P22 monomer	FRET studies	S13
Cowpea Chlorotic Mottle Virus (CCMV)	28	EGFP	K-coil/E-coil recognition	-	S14
Aquifex aeolicus Lumazine Synthase variant (AaLS-13)	~36	GFP (+36)	Electrostatic interaction	-	S15
Aquifex aeolicus Lumazine Synthase (AaLS)	16	GFP-R_{10}	Electrostatic interaction	-	S16
Todarodes pacificus hemocyanin (TpHc)	11 (inner) 16 (height)	GFP	Electrostatic interaction (His-tag GFP binds to Ni^{2+}-NTA-conjugated TpHc)	-	S17

Supplementary Information- Figure S1

Linker 5

MGSDKIHHHHHHMMVISEKVRKALNDQLNREIYSSYLYLSMATYFDAEGFKGFAHWMKKQAQEELTHAMKFYEYI
YERGGRVELEAIEKPPSNWNGIKDAFEAALKHEEFVTQSIYNILELASEEKDHATVSFLKWFVDEQVEEEDQVREILDL
LEKANGQMSVIFQLDRYLGQREPGSPGMVSKGEELFTGVVPILVELDGDVNGHKFSVRGEGEGDATNGKLTLKFICT
TGKLPVPWPTLVTTLGYGVACFARYPDHMKQHDFFKSAMPEGYVQERTISFKDDGTYKTRAEVKFEGDTLVNRIVLK
GIDFKEDGNILGHKLEYNFNSHKVYITADKQKNGIKANFKTRHNVEDGGVQLADHYQQNTPIGDGPVLLPDNHYLS
HQSKLSKDPNEKRDHMVLKERVTAAGITHDMDELYK*

Linker 7

MGSDKIHHHHHHMMVISEKVRKALNDQLNREIYSSYLYLSMATYFDAEGFKGFAHWMKKQAQEELTHAMKFYEYI
YERGGRVELEAIEKPPSNWNGIKDAFEAALKHEEFVTQSIYNILELASEEKDHATVSFLKWFVDEQVEEEDQVREILDL
LEKANGQMSVIFQLDRYLGQREPGSGSPGMVSKGEELFTGVVPILVELDGDVNGHKFSVRGEGEGDATNGKLTLKF
ICTTGKLPVPWPTLVTTLGYGVACFARYPDHMKQHDFFKSAMPEGYVQERTISFKDDGTYKTRAEVKFEGDTLVNRI
VLKGIDFKEDGNILGHKLEYNFNSHKVYITADKQKNGIKANFKTRHNVEDGGVQLADHYQQNTPIGDGPVLLPDNH
YLSHQSKLSKDPNEKRDHMVLKERVTAAGITHDMDELYK*

Linker 9

MGSDKIHHHHHHMMVISEKVRKALNDQLNREIYSSYLYLSMATYFDAEGFKGFAHWMKKQAQEELTHAMKFYEYI
YERGGRVELEAIEKPPSNWNGIKDAFEAALKHEEFVTQSIYNILELASEEKDHATVSFLKWFVDEQVEEEDQVREILDL
LEKANGQMSVIFQLDRYLGQREPGSGSGSPGMVSKGEELFTGVVPILVELDGDVNGHKFSVRGEGEGDATNGKLTL
KFICTTGKLPVPWPTLVTTLGYGVACFARYPDHMKQHDFFKSAMPEGYVQERTISFKDDGTYKTRAEVKFEGDTLVN
RIVLKGIDFKEDGNILGHKLEYNFNSHKVYITADKQKNGIKANFKTRHNVEDGGVQLADHYQQNTPIGDGPVLLPD
NHYLSHQSKLSKDPNEKRDHMVLKERVTAAGITHDMDELYK*

Linker 7α (α-helix)

MGSDKIHHHHHHMMVISEKVRKALNDQLNREIYSSYLYLSMATYFDAEGFKGFAHWMKKQAQEELTHAMKFYEYI
YERGGRVELEAIEKPPSNWNGIKDAFEAALKHEEFVTQSIYNILELASEEKDHATVSFLKWFVDEQVEEEDQVREILDL
LEKANGQMSVIFQLDRYLGQREPLEQLEQMVSKGEELFTGVVPILVELDGDVNGHKFSVRGEGEGDATNGKLTLKFI
CTTGKLPVPWPTLVTTLGYGVACFARYPDHMKQHDFFKSAMPEGYVQERTISFKDDGTYKTRAEVKFEGDTLVNRI
VLKGIDFKEDGNILGHKLEYNFNSHKVYITADKQKNGIKANFKTRHNVEDGGVQLADHYQQNTPIGDGPVLLPDNH
YLSHQSKLSKDPNEKRDHMVLKERVTAAGITHDMDELYK*

TmaFt

MGSDKIHHHHHHMMVISEKVRKALNDQLNREIYSSYLYLSMATYFDAEGFKGFAHWMKKQAQEELTHAMKFYEYI
YERGGRVELEAIEKPPSNWNGIKDAFEAALKHEEFVTQSIYNILELASEEKDHATVSFLKWFVDEQVEEEDQVREILDL
LEKANGQMSVIFQLDRYLGQRE*

Figure S1. Amino acid sequence of the proteins studied for this research: blue for HisTag, black for TmaFt, red for linker (Lx) and green for mGL.

Supplementary Information- Figure S2

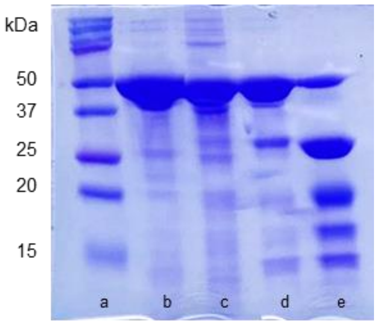

Figure S2. SDS-PAGE gel of a) ladder, b) **7**, c) **5**, d) **9** and e) **7α**

Supplementary Information- Figure S3

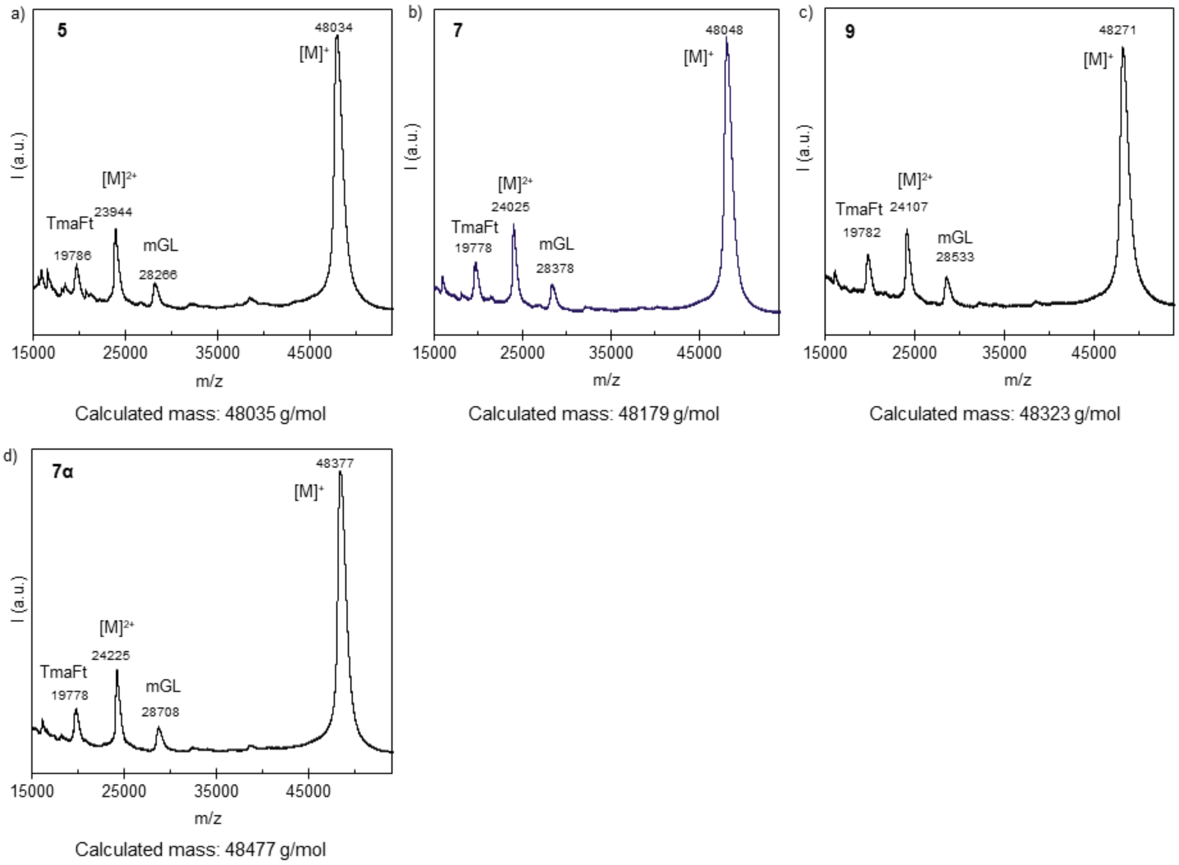

Figure S3. MALDI-TOF results from a) **5**, b) **7**, c) **9** and d) **7α** with the calculated mass for each construct below.

Supplementary Information- Table S2

Table S2. Overview of the excited state lifetimes (τ_{450}) at excitation of 450 nm and photoluminescence quantum yield (ϕ) values of free constructs (left) in solution, mGL@TmaFt cages (middle) at ratio 2:1 in solution, and mGL/**9**/**9**@TmaFT in HPC matrix (right).

Construct	$\tau_{450\ nm}$ (ns)	Φ (%)	Construct within TmaFt	$\tau_{450\ nm}$ (ns)	Φ (%)	Sample	$\tau_{450\ nm}$ (ns)	Φ (%)
mGL	3.2	75	**5**	3.2	71	mGL	4.8	66
**5**	3.2	73	**7**	3.1	71	**9**	3.7	65
**7**	3.1	75	**9**	3.2	72	**9**@TmaFt	3.5	65
**9**	3.2	73	**7α**	3.2	72			
**7α**	3.2	73						

Supplementary Information- Figure S4

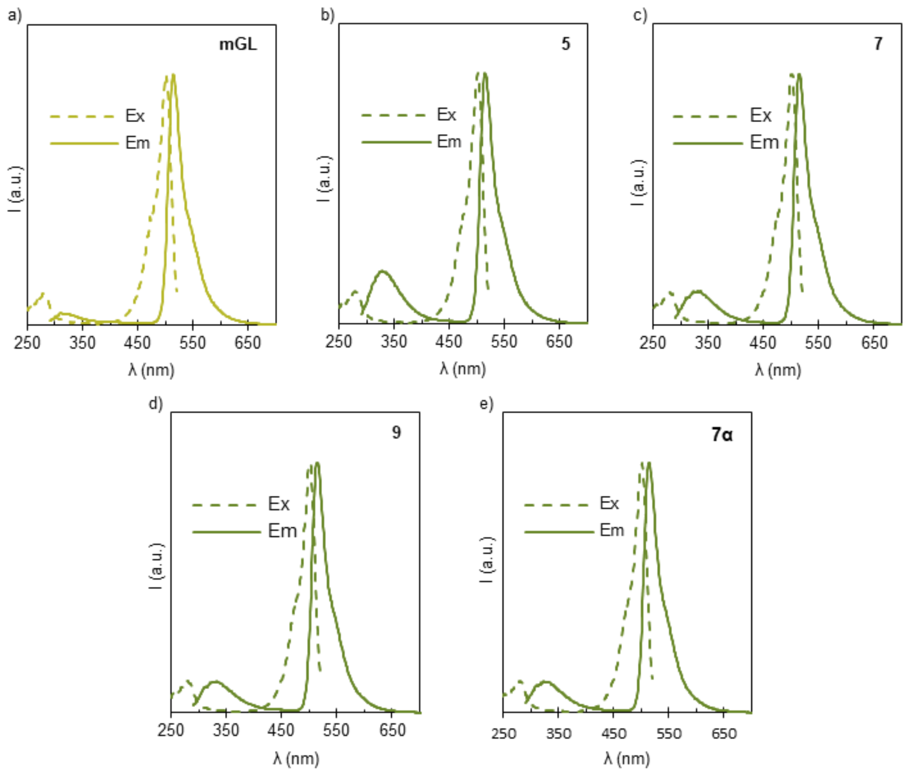

Figure S4. Excitation (dashed line, λ_{em} = 520 nm) and emission (solid line, λ_{exc} = 280 nm) spectra in solution of the proteins a) mGL, b) **5**, c) **7,** d) **9** and d) **7α**

Supplementary Information- Figure S5

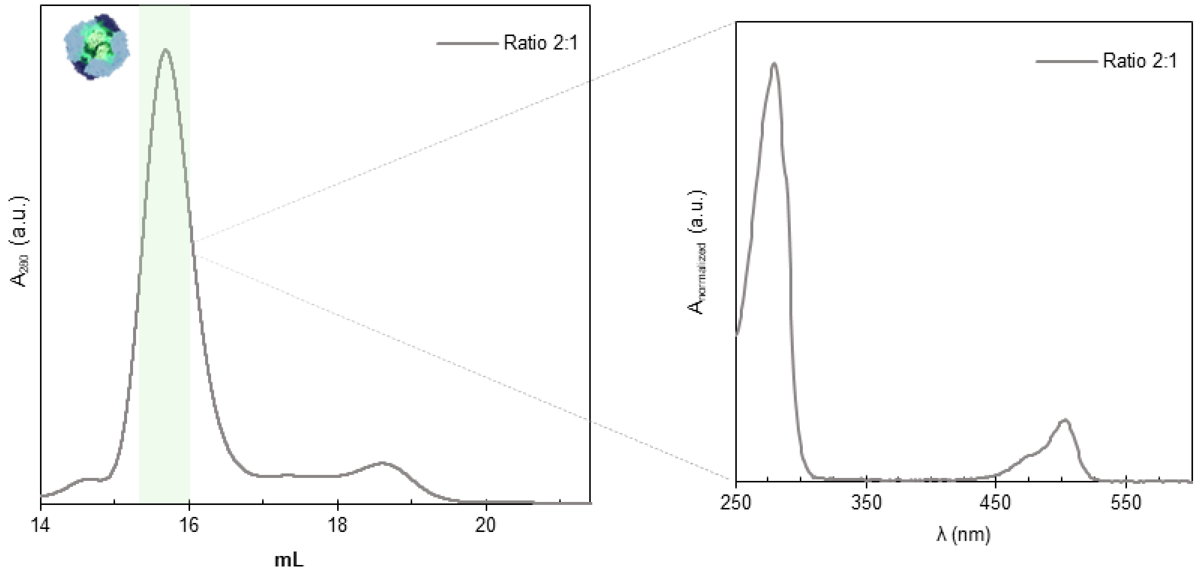

Figure S5. Absorption spectra of the corresponding main peak at 15.7 mL from the SEC carried out for 7@TmaFT at ratio 2:1.

Supplementary Information- Figure S6

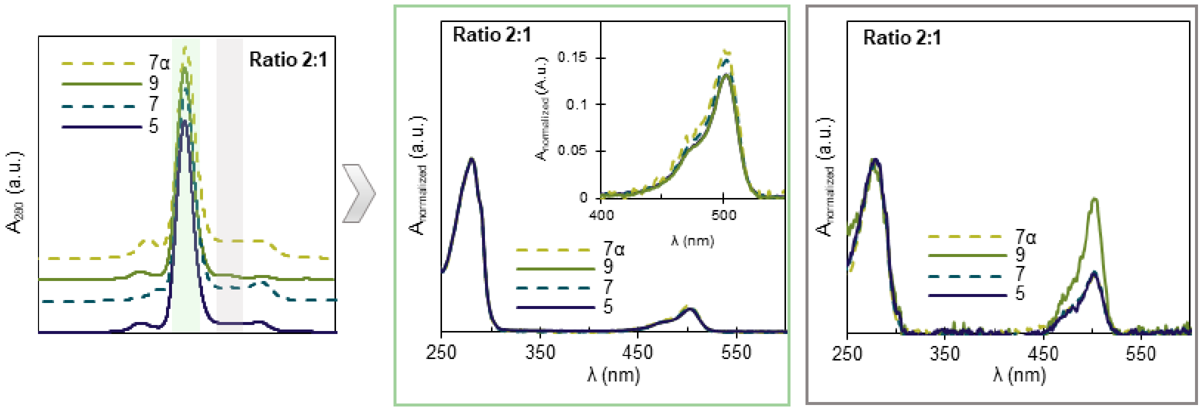

Figure S6. SEC chromatogram at 2:1 ratio for **5**, **7**, **9** and **7α** fusion proteins per cage. Next, the normalized absorption spectra are shown for the main peak at 15.7 mL in green (mGL@TmaFt) and 17.6 mL in grey (mGL-Lx-TmaFt aggregates).

Absorbance was measured for the different fractions collected. Therefore, the assembly yield shown in **Figure 1f** was estimated by dividing the Abs_{503} of the main peak (retention time 15.7 mL corresponding to mGL@TmaFt) by the sum of Abs_{503} for all fractions.

Supplementary Information- Figure S7

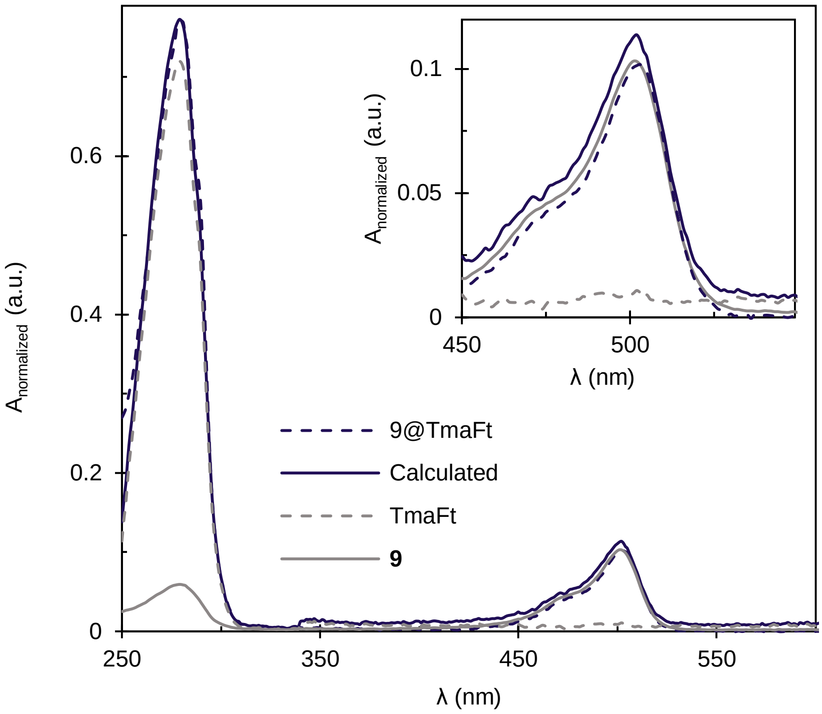

Figure S7. Absorbance spectrum for a concentration of 1 μM.

First, samples were passed through the SEC and fractioned. Absorbance was measured for each fraction. Only those fractions with the sample of interest were used for the following calculations. The concentration of each

sample was determined upon division by their corresponding absorption coefficient ($\varepsilon_{503\ mGL}$ = 101800 $M^{-1}cm^{-1}$ for the FPs, and $\varepsilon_{280\ TmaFt\ monomer}$ = 29910 $M^{-1}cm^{-1}$ for the cages) and normalized to the calculated absorption of 1 μM (**Figure 2c**). The A_{280} of **9** is then subtracted from that of **9**@TmaFt and subsequently divided by the $\varepsilon_{280\ TmaFt}$, determining the concentration of shell and, therefore, the cargo-to-shell ratio.

Supplementary Information- Figure S8

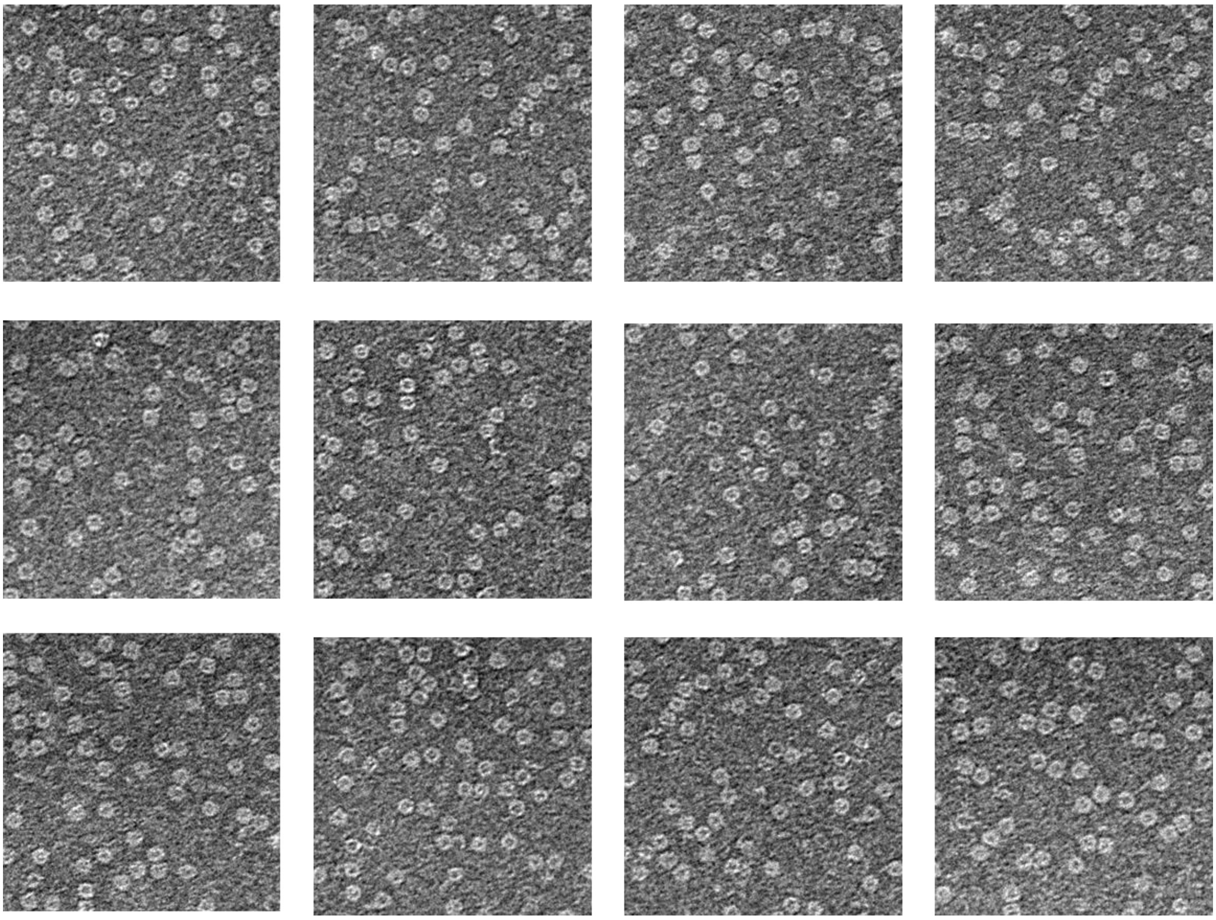

Figure S8. TEM images of **9**@TmaFt at ratio 2:1. Image width corresponds to 200 nm.

Supplementary Information- Figure S9

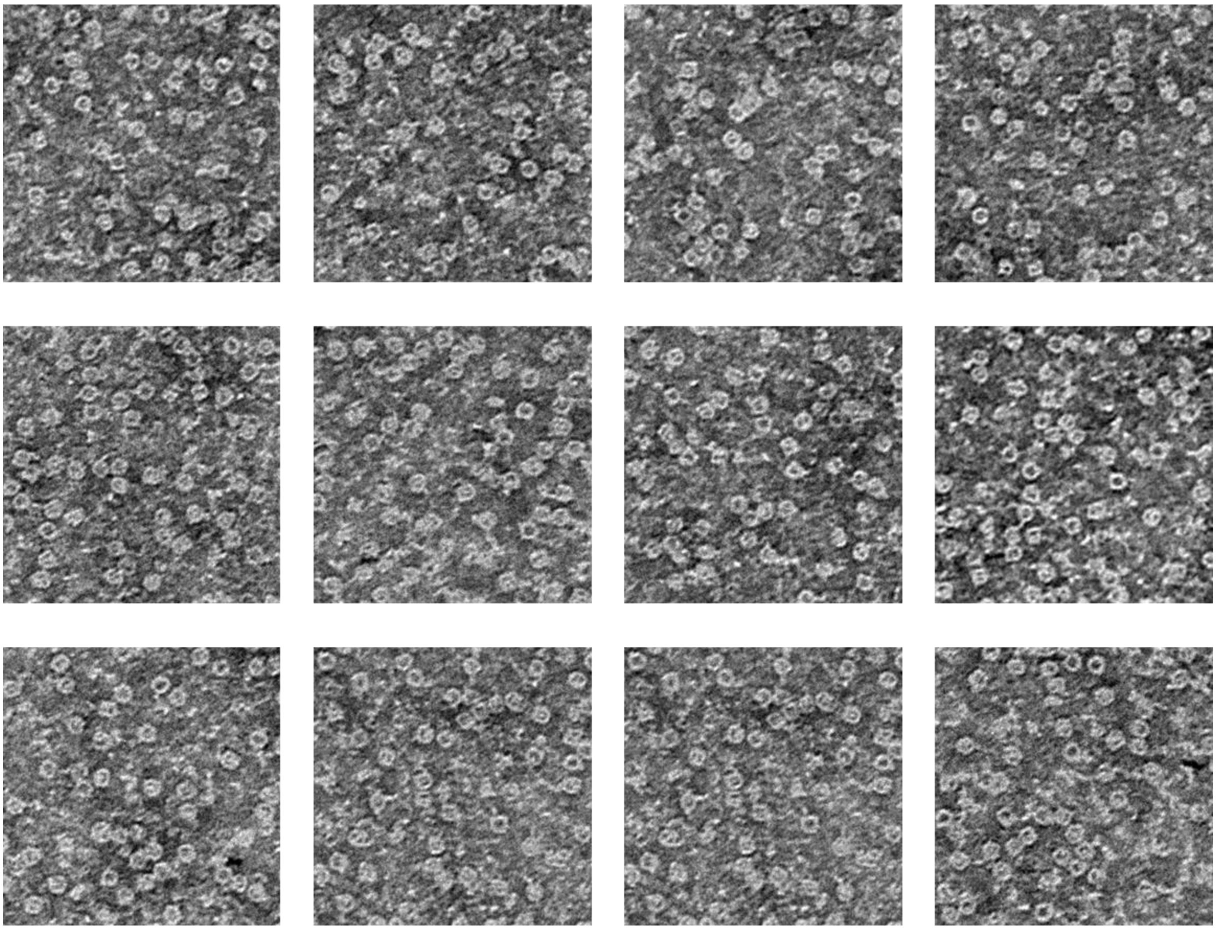

Figure S9. TEM images of **9**@TmaFt at ratio 4:1. Image width corresponds to 200 nm.

Supplementary Information- Figure S10

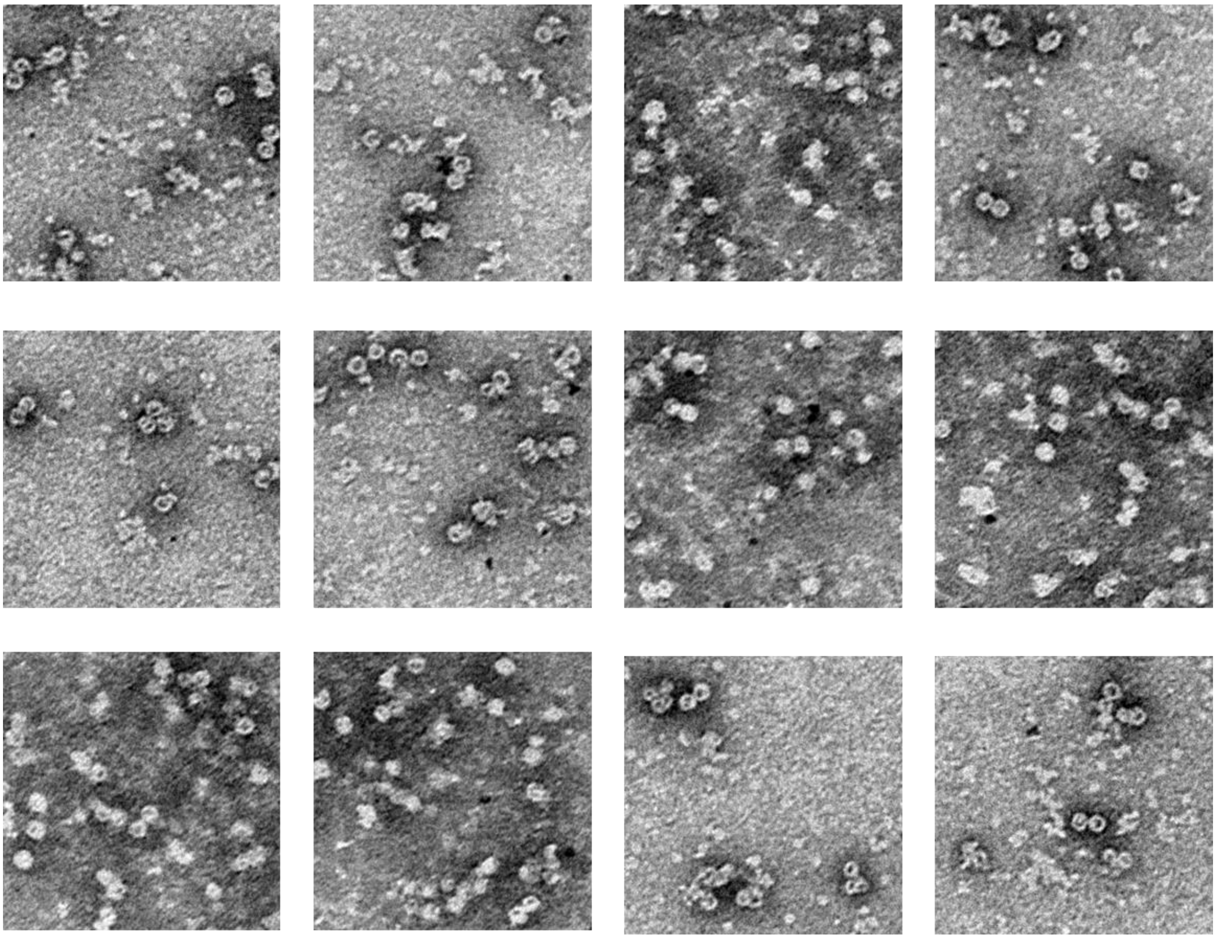

Figure S10. TEM images of **9**@TmaFt at ratio 6:1. Image width corresponds to 200 nm.

Supplementary Information- Figure S11

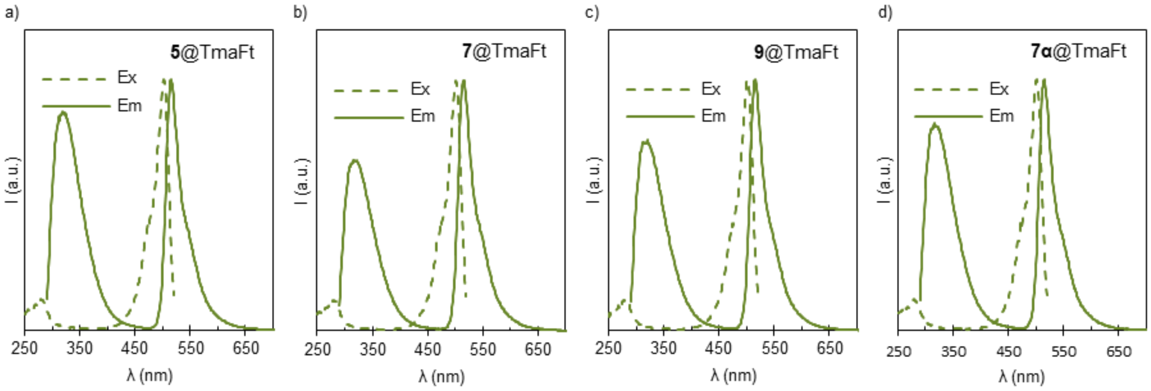

Figure S11. Excitation (dashed line) and emission (solid line) spectra in solution of the mGL@TmaFt, measured at and emission of 520 nm and excitation of 280 nm, respectively.

Supplementary Information- Figure S12

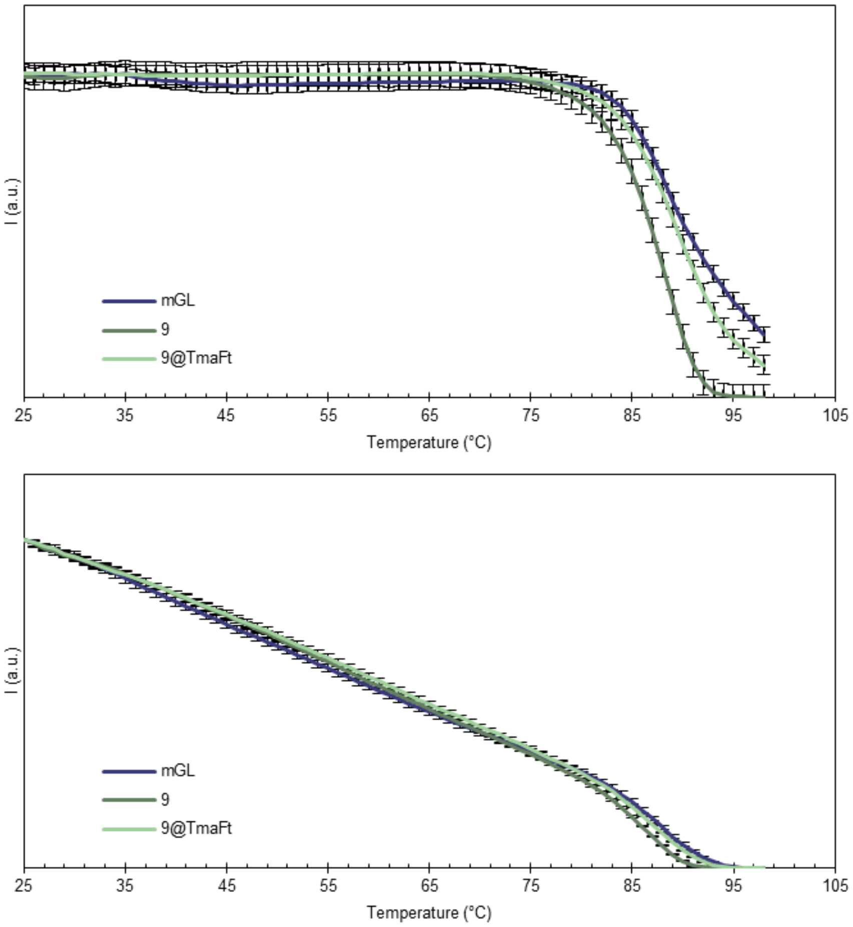

Figure S12. Temperature of non-reversibility (T_{nr}) (top) and melting temperature (T_m) (bottom) in solution of **9**@TmaFt in 2:1 ratio, **9** and mGL.

__Supplementary Information- Figure S13__

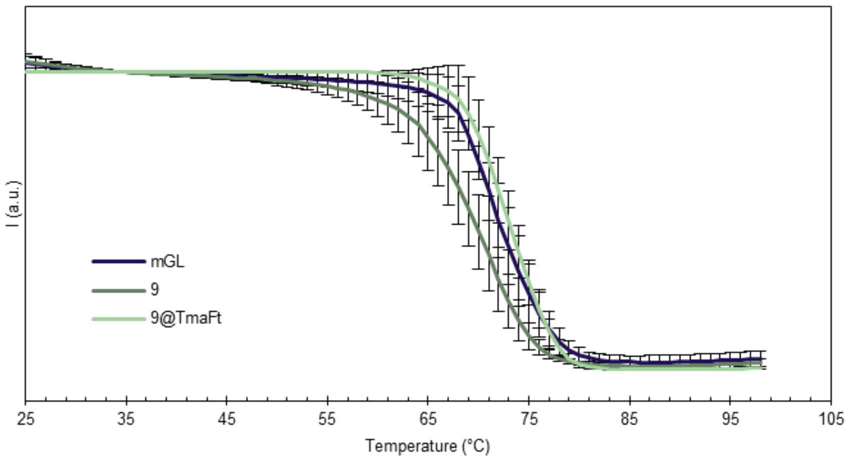

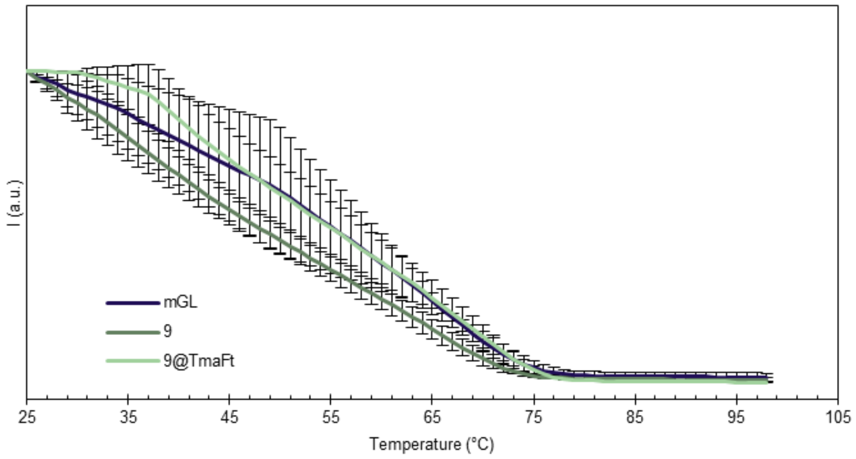

Figure S13. Temperature of non-reversibility (T_{nr}) (top) and melting temperature (T_m) (bottom) in HPC matrix of **9**@TmaFt in 2:1 ratio, **9** and mGL.

Supplementary Information- Figure S14

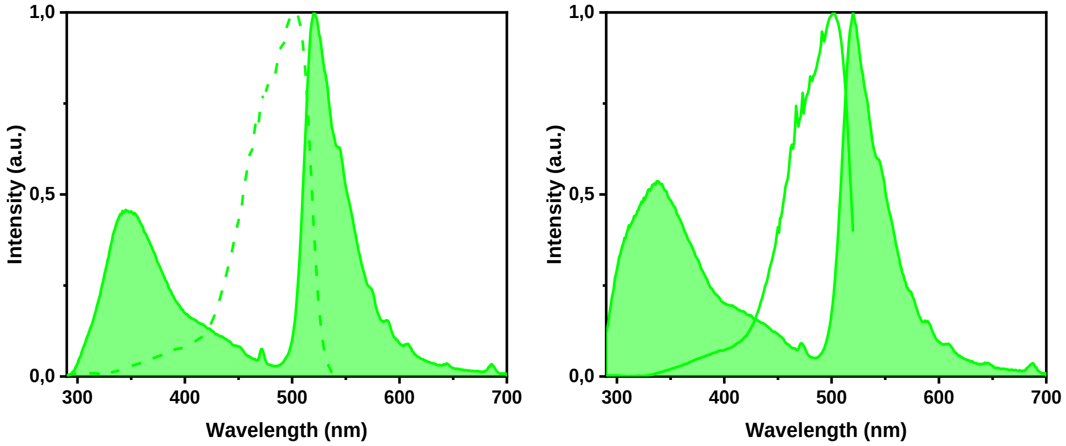

	t_{450} (ns)
1	3.6

	t_{450} (ns)
2	3.5

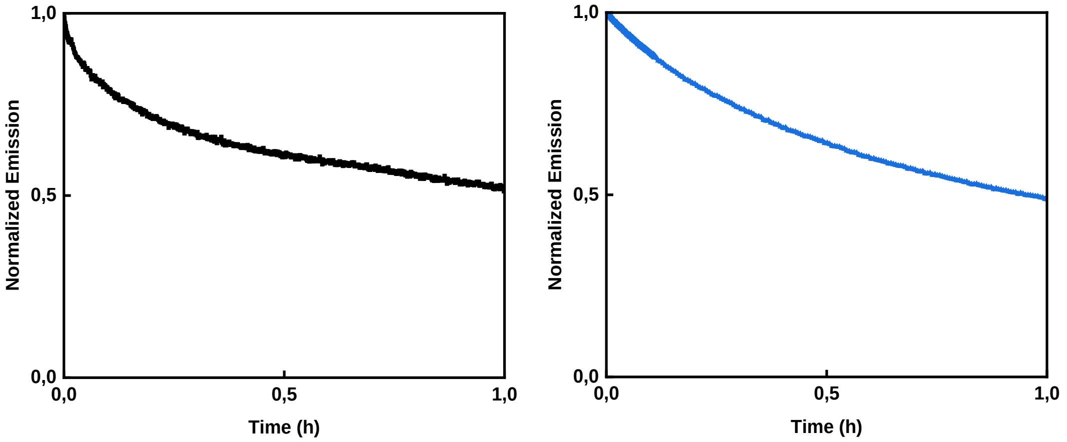

Figure S14. Replicate of the device stability prepared with different batches of **9**@TmaFt highlighted by the emission intensity decay coating over time at 200 mA.

References

S1 S. Chakraborti, A. Korpi, M. Kumar, P. Stępień, M. A. Kostiainen and J. G. Heddle, *Nano Lett.*, 2019, **19**, 3918–3924.

S2 S. Nagano, E. F. Banwell, K. Iwasaki, M. Michalak, R. Pałka, K. Y. J. Zhang, A. R. D. Voet and J. G. Heddle, *Adv. Mater. Interfaces*, 2016, **3**, 1600846.

S3 H. L. Svilenov, T. Menzen, K. Richter and G. Winter, *Mol. Pharmaceutics*, 2020, **17**, 2638–2647.

S4 A. Sillen and Y. Engelborghs, *Photochem Photobiol*, 1998, **67**, 475–486.

S5 X. Han and K. J. Woycechowsky, *Biochemistry*, 2017, **56**, 6211–6220.

S6 J. K. Rhee, M. Hovlid, J. D. Fiedler, S. D. Brown, F. Manzenrieder, H. Kitagishi, C. Nycholat, J. C. Paulson and M. G. Finn, *Biomacromolecules*, 2011, **12**, 3977–3981.

S7 Y. Azuma, R. Zschoche and D. Hilvert, *Journal of Biological Chemistry*, 2017, **292**, 10321–10327.

S8 C. Cassidy-Amstutz, L. Oltrogge, C. C. Going, A. Lee, P. Teng, D. Quintanilla, A. East-Seletsky, E. R. Williams and D. F. Savage, *Biochemistry*, 2016, **55**, 3461–3468.

S9 S. Deshpande, N. D. Masurkar, V. M. Girish, M. Desai, G. Chakraborty, J. M. Chan and C. L. Drum, *Nat Commun*, 2017, **8**, 1442.

S10 K. W. Pulsipher, J. A. Bulos, J. A. Villegas, J. G. Saven and I. J. Dmochowski, *Protein Science*, 2018, **27**, 1755–1766.

S11 S. Tetter and D. Hilvert, *Angewandte Chemie*, 2017, **129**, 15129–15132.

S12 I. J. Minten, L. J. A. Hendriks, R. J. M. Nolte and J. J. L. M. Cornelissen, *J Am Chem Soc*, 2009, **131**, 17771–17773.

S13 A. O'Neil, P. E. Prevelige, G. Basu and T. Douglas, *Biomacromolecules*, 2012, **13**, 3902–3907.

S14 I. J. Minten, R. J. M. Nolte and J. J. L. M. Cornelissen, *Macromol Biosci*, 2010, **10**, 539–545.

S15 B. Wörsdörfer, Z. Pianowski and D. Hilvert, *J Am Chem Soc*, 2012, **134**, 909–911.

S16 F. P. Seebeck, K. J. Woycechowsky, W. Zhuang, J. P. Rabe and D. Hilvert, *J Am Chem Soc*, 2006, **128**, 4516–4517.

S17 T. Hashimoto, Y. Ye, M. Ui, T. Ogawa, T. Matsui and Y. Tanaka, *Biochem Biophys Res Commun*, 2019, **514**, 31–36.
